# Fox smell abrogates the effect of herbal odor to prolong mouse cardiac allograft survival

**DOI:** 10.1186/1749-8090-9-82

**Published:** 2014-05-09

**Authors:** Xiangyuan Jin, Masateru Uchiyama, Qi Zhang, Masanori Niimi

**Affiliations:** 1 Department of Surgery, Teikyo University, 2-11-1 Kaga, Itabashi-ku, Tokyo 173-8605, Japan; 2 Department of Thoracic and Cardiovascular Surgery, the 4th Affiliated Hospital of Harbin Medical University, 37 Yiyuan Street, Nangang District, Harbin, Heilongjiang Province 150001, China; 3 Department of Cardiovascular Surgery, Teikyo University, 2-11-1 Kaga, Itabashi-ku, Tokyo 173-8605, Japan; 4 Department of Dermatology, Huashan Hospital, Fudan University, Urumqi Road 12, Shanghai, China

**Keywords:** Odor, Tokishakuyaku-san, Heart transplantation, Regulatory cells, Mouse

## Abstract

**Background:**

Herbal medicines have unique odors, and the act of smelling may have modulatory effects on the immune system. We investigated the effect of olfactory exposure to Tokishakuyaku-san (TJ-23), a Japanese herbal medicine, on alloimmune responses in a murine model of cardiac allograft transplantation.

**Methods:**

Naïve or olfactory-dysfunctional CBA mice underwent transplantation of a C57BL/6 heart and were exposed to the odor of TJ-23 until rejection. Some naïve CBA recipients of an allograft were given olfactory exposure to Sairei-to (TJ-114), trimethylthiazoline (TMT), individual components of TJ-23, or a TJ-23 preparation lacking one component. Adoptive transfer studies were performed to determine whether regulatory cells were generated.

**Results:**

Untreated CBA mice rejected their C57BL/6 allografts acutely, as did olfactory-dysfunctional CBA mice exposed to the odor of TJ-23. CBA recipients of a C57BL/6 heart given olfactory exposure to TJ-23 had significantly prolonged allograft survival, whereas those exposed to the odor of TJ-114, TMT, one component of TJ-23, or TJ-23 lacking a component did not. Secondary allograft recipients that were given, at 30 days after transplantation, either whole splenocytes, CD4^+^ cells, or CD4^+^CD25^+^ cells from primary recipients exposed to the odor of TJ-23 had indefinitely prolonged allograft survival.

**Conclusions:**

Prolonged survival of cardiac allografts and generation of regulatory cells was associated with exposure to the odor of TJ-23 in our model. The olfactory area of the brain may have a role in the modulation of immune responses.

## Background

Brain function might be correlated with immune resposes [1]. In previous study, we investigated the effects of auditory stimulation of Opera in murine heart transplantation model and confirmed the correlation. Next to auditory stimulation of Opera music and sounds, our current study investigated the effects of olfactory stimulation of herbal medicines on alloimmune responses in murine heart transplantation model.

Odors received through the epithelium of the nose are converted to different topographical maps in the olfactory bulb of the brain and can induce aversion or attraction [2]. Herbal medicines usually have unique scents, and exposure to these odors may affect physiologic functions and clinical symptoms. Thus, aromatherapy is used for pain relief, induction of relaxation, reduction of anxiety, and energy enhancement [3]. Moreover, the olfaction process may have modulatory effects on the immune system [4,5]. However, no study has investigated a possible relationship between smelling and transplantation.

New immunosuppressive drugs have improved allograft survival rates in patients who have undergone transplantation, but long-term outcomes are compromised by graft loss due to chronic rejection and the side effects of nonspecific lifelong immunosuppressive therapy, which increases the risk of infection and malignant disease [6-9]. More research is needed on methods for minimizing the risks associated with transplantation and immunosuppressive therapy.

Studies in experimental models have shown that immunologic tolerance involves both central and peripheral mechanisms [10]. Peripheral tolerance can be achieved by such mechanisms as anergy, deletion, ignorance, and active immune regulation [11,12]. Among the mechanisms of peripheral tolerance, active suppression by regulatory T cells is likely to have a crucial role in maintaining tolerance to transplants [13,14]. Once donor-specific regulatory cells have been induced and survive in the recipient of a graft, it may be possible to modify the lifelong use of nonspecific immunosuppressive agents. Therefore, identification of agents that promote induction and maintenance of regulatory cells may have implications for the development of new tolerogenic strategies in transplantation.

Since Japanese government health officials officially recognized the therapeutic effects of Japanese herbal medicines about 30 years ago, these agents have been widely used as alternative therapy for several diseases. We previously demonstrated the efficacy of several commonly employed agents in inducing donor-specific regulatory cells and prolonging allograft survival in mice [15-21]. Li and Weir [22] showed that Radix Tripterygium wilfordii, a Chinese herbal medicine, has immunosuppressive effects in human blood mononuclear cells in vitro. In recent studies in our murine model, oral administration of the Japanese herbal medicines Sairei-to (TJ-114) [23], Tokishakuyaku-san (TJ-23) [24], or Artemisiae Capillaris Herba [25] was associated with significantly prolonged survival of allogeneic cardiac grafts and generation of regulatory cells. In the current study, we examined whether olfactory exposure to TJ-23 affected duration of allograft survival in the same model.

## Methods

### Mice

Male C57BL/6 (B6, H2^b^), CBA (H2^k^), and BALB/c (H2^d^) mice that were 8 to 12 weeks of age were purchased from Sankyo Ltd (Tokyo, Japan). CBA mice with olfactory dysfunction induced by excision of the olfactory bulb in a stereotaxic procedure were purchased from Charles River Laboratories (Tokyo). All mice were housed in conventional facilities at the Biomedical Services Unit of Teikyo University and used in accordance with the guidelines for animal experimentation approved by the Animal Use and Care Committee of Teikyo University.

### Heart transplantation

All transplant procedures were performed with the mice under general anesthesia. Fully vascularized heterotopic hearts from B6 or BALB/c donors were transplanted into CBA mice by using microsurgical techniques [26]. Postoperatively, graft function was assessed daily by palpation for evidence of contraction. Rejection was defined as complete cessation of the heartbeat and confirmed by direct visualization and histologic examination of the graft.

### Exposure to odors

To accomplish exposure to the odor of an agent, 12.5 g of the agent was added to 500 mL of water in an electric pot placed in a chamber and the mixture was boiled intermittently so that a temperature of 28°C to 30°C and a relatively humidity level of 60% was maintained in the chamber. The agent/water mixture was changed at the same time every day. CBA recipients of a B6 heart were put into the chamber from the day of transplantation until rejection. Within the chamber, mice underwent olfactory exposure to one of the following: water vapor alone (control group); TJ-23; TJ-114; trimethylthiazoline (TMT), which is secreted from the anal gland of fox and induces aversive behavior and fear responses in mice [2]; a mixture of TJ-23 and TMT; each component (Table 1) of TJ-23; or a preparation of TJ-23 from which one of its six components had been removed (Table 2). Olfactory-dysfunctional CBA and olfactory bulb sham-operated CBA recipients of a B6 heart were exposed to the odor of TJ-23. All herbal medicines were gifts from Tsumura (Tokyo, Japan).

**Table 1:** Allograft survival duration in mice given olfactory exposure to individual components of Tokishakuyaku-san

**Component**	**Individual graft survival (d)**	**MST (d)**
Cnidii rhizoma	7, 8, 11, 13, 16	11
Alismatis rhizoma	7, 8, 8, 14, 50	8
Poria sclerotium	7, 8, 8, 9, 10	8
Paeoniae radix	7, 7, 8, 9, 18	8
Angelicae radix	7, 7, 8, 8, 22	8
Atractylodis lanceae rhizome	7, 7, 7, 11, 18	7

MST, median survival time.

**Table 2:** Allograft survival duration in mice given olfactory exposure to either Tokishakuyaku-san with one component removed or to water vapor

**Component removed/water vapor**	**Individual graft survival (d)**	**MST (d)**
Alismatis rhizoma	6, 10, 21, 37, 48	21
Atractylodis lanceae rhizome	7, 9, 13, 16, 19	13
Angelicae radix	7, 9, 12, 14, 16	12
Paeoniae radix	7, 7, 10, 19, 26	10
Cnidii rhizoma	7, 8, 8, 8, 28	8
Poria sclerotium	7, 7, 8, 8, 12	8
Water	6, 6, 6, 7, 8, 9, 9, 16, 20, 20	8.5

MST, median survival time.

### Adoptive transfer studies

Adoptive transfer studies were conducted to determine whether regulatory cells were generated by olfactory exposure to TJ-23. Thus, 30 days after CBA recipients (primary recipients) underwent transplantation of a B6 cardiac allograft and were exposed to the odor of TJ-23, splenocytes (5.0 × 10^7^) from primary recipients with functioning allografts were adoptively transferred into naive CBA mice (secondary recipients). After the adoptive transfer, the secondary recipients underwent transplantation of a B6 or BALB/c heart immediately. In some experiments, CD4^+^ and CD4^+^CD25^+^ cells were purified from the spleens of primary transplant recipients exposed to the odor of TJ-23 by positive selection using a magnetically activated cell sorter (MACS), CD4 microbeads (Miltenyi Biotec, Auburn, CA; purity > 98%), and mouse CD4+ CD25+ regulatory T-cell isolation kit (Miltenyi Biotec). 2.0 × 10^7^ of the CD4^+^ cells or 1.0 × 10^6^ of the CD4^+^CD25^+^ cells were adoptively transferred into naïve secondary recipients, which then immediately underwent transplantation of a B6 heart.

### Immunohistochemical and histologic studies of cardiac grafts

Cardiac allografts in untreated mice and mice exposed to the odor of TJ-23 were removed 30 days after transplantation and studied immunohistochemically with use of double immunostaining. Fresh 4-μm-thick graft cryosections were fixed in ice-cold acetone and preincubated in Block Ace (Dainippon Pharmaceutical Co., Ltd, Tokyo, Japan). Samples were incubated with anti-Foxp3 (kindly provided by Professor Kenjiro Matsuno, Dokkyo Medical University, Tochigi, Japan) polyclonal antibody; incubated with alkaline phosphatase (ALP)-conjugated anti-rabbit Ig (712-055-152; Jackson ImmunoResearch Laboratories, West Grove, PA, USA) for anti-Foxp3; and developed blue with Vector Blue (Vector Laboratories, Burlingame, CA). Cryosections were then incubated with rabbit anti-mouse type IV collagen polyclonal antibody (LB1403; Cosmo Bio, Tokyo) and peroxidase-conjugated anti-rabbit Ig (55693; Mitsubishi Chemical, Tokyo) and then developed brown with diaminobenzidine (Vector Laboratories).

Cardiac allografts in untreated mice and mice exposed to the odor of TJ-23 were removed 30 days after transplantation and studied histologically. Frozen sections (4-μm thick) were cut, mounted on silane-coated slides, and stained with hematoxylin-eosin.

### Flow cytometry analysis

Thirty days after cardiac allograft transplantation, splenocytes from recipients exposed to the odor of TJ-23 and untreated recipients were stained with fluorochrome-conjugated anti-CD4 or anti-CD25 monoclonal antibody (mAb) (RM4-5 and PC61, respectively; BD Biosciences, San Jose, CA, USA) and antimouse Foxp3 mAb (FJK-16s; eBioscience, San Diego, CA), as well as their isotype controls (eBioscience). The stained cells were analyzed by using a FACS Canto2 system (BD Biosciences), and the percentage of CD4^+^CD25^+^Foxp3^+^ in CD4^+^ cells was determined.

### Mixed leukocyte culture (MLC) studies and cytokine assays

In MLC studies [27], the responder cells were splenocytes from naïve CBA mice, untreated mice, or CBA mice exposed to the odor of TJ-23 that had undergone transplantation of a B6 heart 14 days earlier. Proliferation was assessed by using an Enzyme-linked Immunosorbent Assays (ELISA) for bromodeoxyuridine incorporation (Biotrak, version 2, Amersham, Little Chalfont, United Kingdom [UK]) according to the manufacturer’s instructions. MLC studies were performed as described previously [25].

Enzyme-linked immunoabsorbent assays (ELISAs) were performed as described previously [25] to assess plasma levels of interleukin (IL)-2, IL-4, IL-10, and interferon (IFN)-γ in the supernatant of the MLC on day 4.

### Statistical analysis

Cardiac allograft survival in groups of mice was compared by using Mann-Whitney U tests (Graphpad Prism; San Diego, CA, USA). In the cell-proliferation, cytokine studies, and flow cytometry studies, two groups were compared by using unpaired Student *t* tests (Graphpad Prism). A *P* value of less than 0.05 was considered to represent a significant difference between groups.

## Results

### Effect of various odors on survival of cardiac allografts

**Figure 1:**
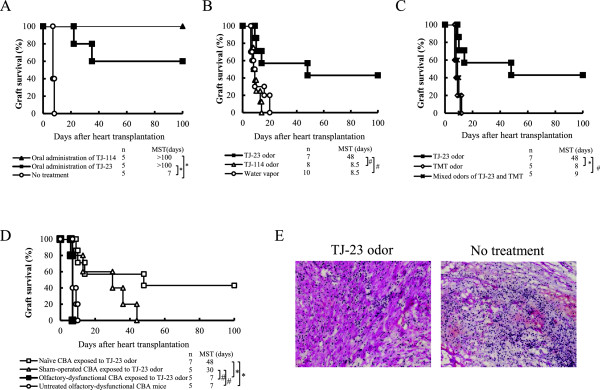
**Allograft survival of CBA mice given oral administration or exposed to various odors of Japanese Herbal Medicines and histologic findings in CBA mice. (A)** Results in recipients of a C57BL/6 heart that were untreated or given oral administration of TJ-23 and TJ-114 from the day of transplantation until 7 days afterward. MST, median survival time; *P < 0.01 for difference between 2 groups. **(B)** Results in recipients of a C57BL/6 heart that were exposed to either water vapor alone or to the odors of TJ-23 and TJ-114 from the day of transplantation until allograft rejection. MST, median survival time; #*P* < 0.05 for difference between two groups. **(C)** Results in recipients of a C57BL/6 heart that were exposed to the odor of TJ-23, trimethylthiazoline (TMT), or both agents from the day of transplantation until allograft rejection. MST, median survival time; #*P* < 0.05 and *P < 0.01 for difference between two groups. **(D)** Results in naïve, olfactory bulb sham-operated and olfactory-dysfunctional CBA recipients of a C57BL/6 heart exposed to the odor of TJ-23 and in olfactory-dysfunctional CBA recipients with no exposure. Mice were treated from the day of transplantation until allograft rejection. MST, median survival time; *P < 0.01 and #*P* < 0.05 for difference between two groups. **(E)** Histologic studies of cardiac allografts obtained from mice given olfactory exposure to TJ-23 and untreated mice (hematoxylin and eosin stain; magnification × 40).

Our previous studies showed that the majority of CBA recipients given oral administration of TJ-114 and TJ-23 indefinitely prolonged cardiac allograft survival while untreated recipients rejected allograft acutely (median survival times [MSTs], >100, >100 and 7days, respectively; *P* < 0.01; Figure 1A).

CBA mice that were given a B6 cardiac graft and underwent olfactory exposure to either water vapor or TJ-114 rejected their allografts acutely (MST, 8.5 days for both exposures; Figure 1B). In contrast, allografts in mice exposed to the odor of TJ-23 had significantly prolonged survival duration (MST, 48 days; individual allograft survival times, 9, 10, 14, 48, > 100, > 100, and > 100 days; *P* < 0.05 vs either the water-exposed or TJ-114-exposed group; Figure 1B). None of the individual components of TJ-23 had this effect (Table 1), nor did any of the preparations of TJ-23 with one component removed (Table 2).

CBA recipients of allografts that were exposed to the odor of TMT (fox smell) rejected their grafts acutely (MST, 8 days; *P* < 0.01 vs TJ-23-exposed group; Figure 1C), whereas those exposed to a mixture of the odors of TJ-23 and TMT had significantly shorter allograft survival compared with the TJ-23-exposed group (MST, 9 days; *P* < 0.05; Figure 1C).

### Effect of olfactory dysfunction on allograft survival

CBA mice in which the olfactory bulb had been excised rejected their B6 grafts acutely, even if they had been exposed to the odor of TJ-23 (MST, 7 days for both untreated and TJ-23-exposed mice; *P* < 0.01 vs naïve TJ-23-exposed mice for both comparisons; Figure 1D). When B6 hearts were transplanted into olfactory bulb sham-operated CBA mice exposed to the odor of TJ-23, the allograft survival was prolonged significantly (MST, 30 days; *P* < 0.05 vs untreated and TJ-23-exposed olfactory dysfunction mice).

### Histologic features of allografts

Histologic examinations of cardiac allografts obtained 30 days after transplantation from mice given olfactory exposure to TJ-23 showed some cell infiltration, preserved graft structure, and mild myocardial injuries. Allografts from untreated recipients showed severe myocyte damage, edema, and aggressive cell infiltration characteristic of the acute rejection process (Figure 1E).

### Generation of regulatory cells in mice given olfactory exposure to TJ-23

**Figure 2:**
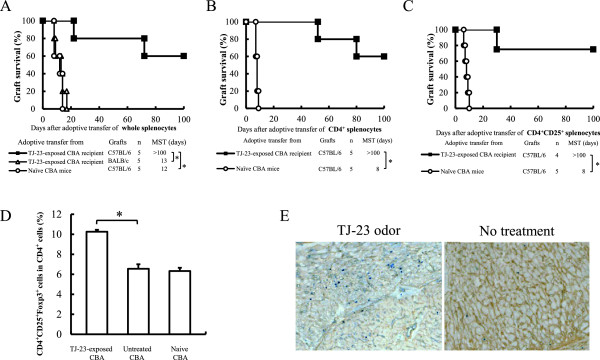
**Evidence of generation of regulatory cells in CBA recipients of cardiac allografts given olfactory exposure to TJ-23.** Allograft survival times after adoptive transfer of whole splenocytes **(A)**, CD4^+^ splenocytes **(B)**, or CD4^+^CD25^+^ splenocytes **(C)**. MST, median survival time; *P < 0.01 for difference between two groups. **(D)** CD4, CD25, and Foxp3 expression in splenocytes, as determined by flow cytometry. Data are mean ± SD values for the percentage of CD4^+^CD25^+^Foxp3^+^ in CD4^+^ cells in samples from five mice in each group. *P < 0.01 for difference between 2 groups. **(E)** Foxp3^+^ cells detected by double immunostaining in cardiac allografts obtained 30 days after transplantation from mice given olfactory exposure to TJ-23 and untreated mice (magnification × 40).

In the adoptive transfer studies, secondary CBA recipients of a B6 heart that were given whole splenocytes from primary TJ-23-exposed CBA recipients of a B6 cardiac graft 30 days after grafting had indefinitely prolonged survival of their allografts (MST, > 100 days; Figure 2A). The MSTs of allografts in secondary recipients given splenocytes from either naïve CBA mice or from third-party (BALB/c) mice were significantly shorter (MST, 12 and 13 days, respectively; *P* < 0.01 vs TJ-23-exposed secondary recipients; Figure 2A).

Secondary CBA recipients of a B6 heart that were given either CD4^+^ or CD4^+^CD25^+^ cells purified from the spleens of primary TJ-23-exposed CBA recipients of a B6 heart had indefinitely prolonged survival of their allografts (MST, > 100 days for each cell type; *P* < 0.01 vs both CD4^+^ and CD4^+^CD25^+^ controls [ie, adoptive transfer of cells from naïve CBA mice]; Figure 2B and 2C). In contrast, the MST of B6 allografts in secondary CBA recipients that underwent adoptive transfer of either CD4^+^ or CD4^+^CD25^+^ cells from the spleens of naïve CBA mice was 8 days. These data indicate that olfactory exposure to TJ-23 generated regulatory cells which might be donor specific in the primary recipients and that one of the regulatory populations consisted of CD4^+^CD25^+^ cells.

Flow cytometry studies showed that the population of CD4^+^CD25^+^Foxp3^+^ cells in the CD4^+^ cells was increased in the spleens of CBA recipients of allografts exposed to the odor of TJ-23 compared with those of untreated recipients (*P* < 0.01; Figure 2D). Immunohistochemical studies showed that cardiac allografts from TJ-23-exposed recipients had more Foxp3^+^ cells than those from untreated mice (Figure 2E). These data suggest that the CD4^+^ regulatory cells contained a population that was CD4^+^CD25^+^Foxp3^+^.

### Cell proliferation and cytokine production in mice given olfactory exposure to TJ-23

**Figure 3:**
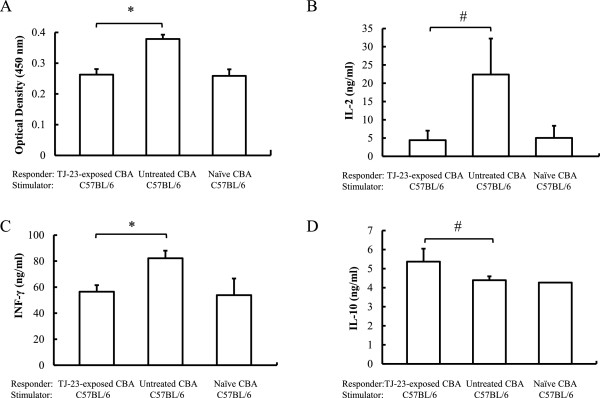
**Evidence of induction of alloproliferative hyporesponsiveness in CBA recipients of allograft exposed to the odor of TJ-23. (A)** Results of cell-proliferation assays in mixed leukocyte cultures (MLCs). Data are mean ± SD values derived from samples from five mice in each group. *P < 0.01 for difference between two groups. Levels of interleukin (IL)-2 **(B)**, interferon (IFN)-γ **(C)**, and IL-10 **(D)** in the MLCs, as assessed by enzyme-linked immunosorbent assays. Data are shown as mean ± SD values derived from samples from five mice in each group. #*P* < 0.05 and *P < 0.01 for difference between two groups.

Maximum proliferation of naïve CBA splenocytes (responder cells) against B6 splenocytes (stimulator cells) treated with mitomycin C occurred on day 4 of the MLCs. Proliferation of splenocytes from CBA recipients of allografts given olfactory exposure to TJ-23 was significantly suppressed compared with that of splenocytes from untreated recipients (Figure 3A; *P* < 0.01).

Levels of interleukin IL-2 (Figure 3B) and IFN-γ (Figure 3C) in splenocytes from allograft recipients exposed to the odor of TJ-23 were significantly lower than those in splenocytes from recipients not exposed to TJ-23, whereas levels of IL-10 (Figure 3D) were higher. There was no difference between the two groups in levels of IL-4 (data not shown).

## Discussion

In this study, we found that olfactory exposure to TJ-23—but not to a component of TJ-23, a preparation of TJ-23 with one component missing, or both TJ-23 and fox smell—induced hyporesponsiveness to fully mismatched cardiac allografts in mice. The increase in the duration of allograft survival has several possible mechanisms. One is that exposure to the odor of TJ-23 generated regulatory cells. Active suppression by regulatory cells has been found to be an important mechanism of induction and maintenance of self-tolerance [28-34], unresponsiveness to allografts [11], and prevention of vasculopathy in cardiac allografts [35,36]. In our study, adoptive transfer of whole splenocytes from primary CBA recipients given olfactory exposure to TJ-23 induced indefinitely prolonged survival of B6 cardiac allografts in secondary recipients, whereas secondary CBA recipients of a B6 heart given splenocytes from BALB/c mice rejected their allografts acutely. These findings indicate that the exposure to TJ-23 generated regulatory cells that may have been donor specific. In addition, adoptive transfer of CD4^+^ or CD4^+^CD25^+^ splenocytes from primary recipients exposed to the odor of TJ-23 induced indefinitely prolonged survival of allografts in secondary recipients. Moreover, flow cytometry analysis found that the percentage of CD4^+^CD25^+^Foxp3^+^ cells in CD4^+^ cells was increased in TJ-23-exposed allograft recipients compared with untreated recipients. These results confirmed that the regulatory population generated by olfactory exposure to TJ-23 contained CD4^+^CD25^+^ cells.

A second possible mechanism for the effects produced by olfactory exposure to TJ-23 was that the treatment changed the balance between Th-1 and Th-2 cytokines. In TJ-23-exposed mice, the expression of Th-1 cytokines (IL-2 and IFN-γ) was decreased and that of a Th-2 cytokine (IL-10) was increased. IL-10 promotes generation of regulatory T cells in vivo [37] and is required for regulatory T cells to mediate tolerance to alloantigens [38]. Moreover, alloantigen-specific regulatory T cells have been shown to prevent rejection of transplanted organs that is initiated by CD4^+^CD25^+^ T cells [39,40]. Therefore, we suggest that olfactory exposure to TJ-23, which suppressed production of Th1 cytokines in our model, may have protected myocardial cells and induced CD4^+^CD25^+^ regulatory cells by means of up-regulation of Th2 cytokines.

A third mechanism for the prolongation of allograft survival (MST, 48 days) in our model may have been that the unique odor of TJ-23 affected the brain through the olfactory function, thereby modulating the immune system of the transplant recipients. We have already assumed the correlation between stimulation to brain and immune system, and we have demonstrated that auditory stimulation through the brain can induce prolongation of allograft survival and maintain generation of regulatory CD4^+^CD25^+^ cells [1]. In the current study, olfactory-dysfunctional mice exposed to the odor of TJ-23 rejected their grafts acutely (MST, 7 days), however, naïve and olfactory bulb sham-operated CBA mice exposed to the odor of TJ-23 prolonged the allograft survival significantly (MSTs, 48 and 30 days), indicates that the olfactory area of the brain may play an important role in modulation of immune responses. On the other hand, adding the odor of TMT (fox smell) to that of TJ-23 attenuated the prolongation of TJ-23-induced allograft survival (MST, 9 days). Therefore, exposure to the unique odor of TJ-23—but not to a component of TJ-23 or a preparation of TJ-23 with one component missing—may be necessary to induce hyporesponsiveness to fully mismatched cardiac allografts, and the addition of TMT also changes the odor in chamber, results in attenuation of effect on allograft survival.

Finally, the hyporesponsiveness in our model may have resulted from absorption of TJ-23 directly through the respiratory tract or skin rather than via the brain. However, if the effects observed in TJ-23-exposed mice had resulted from transtracheal or percutaneous exposure to TJ-23, our findings with respect to allograft survival in olfactory-dysfunctional recipients would probably have been similar to those in allograft recipients with a normal sense of smell.

## Conclusions

These findings demonstrated that olfactory exposure to TJ-23 induced prolongation of B6 cardiac graft survival and generated CD4^+^CD25^+^Foxp3^+^ regulatory cells in naïve CBA mice but not in CBA mice with olfactory dysfunction. There may be relationships among the unique odor TJ-23; brain function, including olfactory function; and the immune system.

## Abbreviations

ELISA: Enzyme-linked immunosorbent assay; Foxp3: Forkhead box P3; HE: Hematoxylin and Eosin; IFN: Interferon; IL: Interleukin; MST: Median survival time; MLC: Mixed leukocyte culture; MMC: Mitomycin C; mAb: Monoclonal antibody; Th: Helper T cell; TMT: Trimethylthiazoline.

## Competing interests

The authors declare that they have no competing interests.

## Author’s contributions

MN, XJ, and MU participated in research design, XJ and QZ carried out the experiments, MN, XJ and MU participated in the writing of the manuscript, and XJ and MU participated in data analysis. All authors read and approved the final manuscript.
